# Implementation of immunochemical faecal occult blood test in general practice: a study protocol using a cluster-randomised stepped-wedge design

**DOI:** 10.1186/s12885-016-2477-9

**Published:** 2016-07-11

**Authors:** Jakob Søgaard Juul, Flemming Bro, Nete Hornung, Berit Sanne Andersen, Søren Laurberg, Frede Olesen, Peter Vedsted

**Affiliations:** Research Unit for General Practice, Department of Public Health, Aarhus University, Bartholins Allé 2, 8000 Aarhus C, Denmark; Research Centre for Cancer Diagnosis in Primary Care, Department of Public Health, Aarhus University, Bartholins Allé 2, 8000 Aarhus C, Denmark; Department of Clinical Biochemistry, Regional Hospital of Randers, Skovlyvej 1, 8930 Randers NE, Denmark; Department of Public Health Programs, Regional Hospital of Randers, Skovlyvej 1, 8930 Randers NE, Denmark; Department of Surgery, Aarhus University Hospital, Tage Hansens Gade 2, 8000 Aarhus C, Denmark

**Keywords:** Colorectal cancer, General practice, iFOBT, FIT, Faecal occult blood test, Early diagnosis, Symptoms, Denmark

## Abstract

**Background:**

Colorectal cancer is a common malignancy and a leading cause of cancer-related death. Half of patients with colorectal cancer initially present with non-specific or vague symptoms. In the need for a safe low-cost test, the immunochemical faecal occult blood test (iFOBT) may be part of the evaluation of such patients in primary care. Currently, Danish general practitioners have limited access to this test. The aim of this article is to describe a study that will assess the uptake and clinical use of iFOBT in general practice. Furthermore, it will investigate the diagnostic value and the clinical implications of using iFOBT in general practice on patients presenting with non-alarm symptoms of colorectal cancer.

**Methods/Design:**

The study uses a cluster-randomised stepped-wedge design and is conducted in the Central Denmark Region among 836 GPs in 381 general practices. The municipalities of the Region and their appertaining general practitioners will be included sequentially in the study during the first 7 months of the 1-year study period. The following intervention has been developed for the study: a mandatory intervention providing all general practitioners with a starting package of 10 iFOBTs, a clinical instruction on iFOBT use in general practice and online information material from the date of inclusion, and an optional intervention consisting of a continuous medical education on colorectal cancer diagnostics and use of iFOBT.

**Discussion:**

This study is among the first and largest trials to investigate the diagnostic use and the clinical value of iFOBT on patients presenting with non-alarm symptoms of colorectal cancer. The findings will be of national and international importance for the future planning of colorectal cancer diagnostics, particularly for ‘low-risk-but-not-no-risk’ patients with non-alarm symptoms of colorectal cancer.

**Trial registration:**

A Trial of the Implementation of iFOBT in General Practice NCT02308384. Date of registration: 26 November 2014

## Background

Colorectal cancer (CRC) is the second most common type of cancer in Denmark and is a leading cause of cancer-related death [1, 2]. In Denmark, approx. 25 % of all new CRC cases in 2013 were diagnosed in stage IV with a 5-year survival of less than 5 % [3]. Considering that CRC is a potentially curable disease when found in earlier stages and that survival is strongly related to stage at diagnosis, these figures underline the importance of increasing the proportion of CRCs diagnosed in early stages.Several initiatives have been made to support a stage shift towards earlier diagnosis of CRC. One important step is screening. Studies show that screening for CRC using faecal occult blood tests may reduce CRC mortality in the screened age group [4–6]. Despite the advantages of screening, 75–80 % of CRC cases must still be found through symptomatic presentation in general practice [7, 8]. Therefore, another important strategy has been to support urgent referral and investigation of patients with CRC alarm symptoms [9–11]. This initiative provides the general practitioner (GP) with the opportunity to refer patients presenting with alarm symptoms of cancer to an urgent colonoscopy. Alarm symptoms include rectal bleeding, change in bowel habits, iron-deficiency anaemia, weight loss and abdominal pain [12]. However, the positive predictive values (PPVs) of alarm symptoms are low (2–8 %). Thus, the GPs must each refer 10–20 patients with alarm symptoms for further diagnostic workup to catch one person with CRC [13–15].

Screening and urgent referral for alarm symptoms are two important improvements of CRC diagnosis. However, 50 % of CRC cases will present in general practice with vague or non-specific symptoms that are not eligible for urgent referral [16]. These symptoms are most often caused by benign conditions and can be considered as ‘low-risk-but-not-no-risk’ symptoms [17]. Studies indicate that patients presenting these symptoms may have a longer diagnostic interval and progress into advanced cancer stages [18–22]. It is a challenge for the GP to identify the few CRC cases among all the patients with similar symptoms, but without CRC. However, a recent study has shown that CRC patients tend to see their GP more often and have more tests performed than the average patient in the year preceding a CRC diagnosis [23]. This may indicate a potential diagnostic window for early identification of patients with CRC. Thus, tools that can assist the GP in the diagnostic workup of patients presenting with uncharacteristic symptoms of CRC are highly needed.

Immunochemical Faecal Occult Blood Test (iFOBT) may be one solution to the problem. Unlike the guaiac faecal occult blood test (gFOBT), which requires three tests, the iFOBT requires only one test and no pre-test dietary restrictions are needed as the test uses antibodies specific to human globin [24]. Furthermore, studies have found iFOBT to be diagnostically superior to gFOBT [25, 26]. The diagnostic performance of iFOBT has mainly been investigated in relation to screening. In these studies, the sensitivity of iFOBT has generally been found to be 80–90 %, the specificity to be above 90 % and the PPV to be better than for most alarm symptoms (10 %) [26–30]. Only few recent small-scale studies have investigated the use of iFOBT on symptomatic patients, and these findings suggest that iFOBT could be beneficial as a case-finding tool in the detection of CRC in general practice [31–37]. No study has focused on the use of iFOBT in patients presenting with non-alarm symptoms of CRC. Large-scale controlled studies are needed to investigate if and how iFOBT can be used in the diagnostic workup of symptomatic patients in primary care. This paper presents a study that implements iFOBT as a diagnostic tool in general practice in individuals presenting with non-alarm symptoms of CRC.

## Methods/Design

### Aim

The aims of the study are to:

1. Evaluate the uptake and clinical use of iFOBT in general practice after targeted courses for GPs in correct use of the test.
2. Estimate the diagnostic value of iFOBT when used on patients presenting with non-alarm symptoms of CRC.
3. Investigate the clinical implications of using iFOBT for case finding in general practice.

### Setting and study population

The study will be performed in the Central Denmark Region (CDR), which is one of five regions in Denmark. The CDR covers approx. 1.2 million inhabitants, 381 general practices, 836 GPs and 19 municipalities. In each municipality, the GPs are organised in units (except for the GPs in the island municipality of Samsø that are included in the unit of Aarhus). Each unit is headed by a chairman who represents the GPs of the municipality. The GPs in Denmark own their own clinic, and approx. 1550 persons are listed per GP. Clinics operate as either solo practices or partnership practices. Remuneration is based on a mix of capitation (25 %) and fee-for-service (75 %) based on a centrally negotiated collective agreement. The GP acts as a gatekeeper to specialised care in the secondary sector, and the citizens must contact the GP for medical advice unless in case of emergency. Approx. 10–20 % of all consultations in general practice ends up with a referral [38]. Nearly all citizens (99 %) are listed with a specific general practice, and the GP is remunerated on the basis of a contract with Danish Regions and must fulfil certain requirements for waiting time and access. iFOBT is available to Danish GPs only to a limited extent as part of the urgent referral for non-specific serious symptoms that might be cancer [39]. However, no logistic setup is available in general practice regarding ordering and analysing the test.

The study population constitutes the 836 GPs in the 381 general practices in the CDR and the individuals aged 30 years or more who are listed with these practices.

### Design

The study uses a cluster-randomised stepped-wedge design [40]. This design allows us to roll out the study in large scale and to include all general practices in the CDR.

**Fig. 1:**
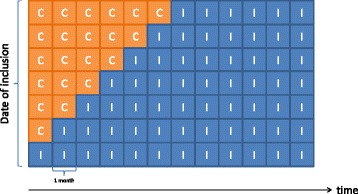
Stepped-wedge design used for the study. Most municipalities start out as usual care (C). Within the first seven months, all will cross over to intervention (I). Seven possible dates of inclusion are available; the 19 municipalities are randomly distributed between these

**Fig. 2:**
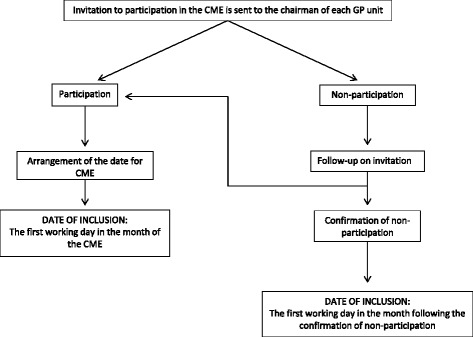
Flowchart of inclusion date for the participating municipalities

During the first seven months of the study period, each municipality and their appertaining GPs will be randomly and stepwise included in the study to receive intervention (Fig. 1). The intervention (see details later) consists of sending iFOBT kits and a clinical guideline to the GPs and to offer an optional continuous medical education (CME) session about CRC diagnosis. The invitation to the CME is sent to the chairman of the GP unit who will arrange the date and time of the meeting. The month in which the CME is arranged determines the date of inclusion for each municipality. Thus, the date at which a municipality is included is defined as the first working day of the month in which the CME is planned to be conducted. As the CME component is optional, the GP units can choose not to participate in the CME. These municipalities are included on the first working day in the month after confirmation of non-participation (Fig. 2). The specific date of the CME is flexibly arranged as the CME can be scheduled to take place on any of the first 7 months, depending on the preferences of the GPs in the municipality (in consideration of other arrangements targeting GPs in the municipality, availability of venue, etc.).

### Randomisation

The study uses cluster randomisation. The 19 municipalities in the CDR are randomly allocated to monthly starts of the intervention, ensuring that all municipalities are included within 7 months. The randomisation is performed prior to initiation of the study and determines when each cluster is offered to participate in the CME. The randomisation is blinded to the research group and is manually performed by two research fellows with no connection to the project.

### Intervention

The intervention was developed using the behavioural change wheel as analytical framework to identify potential barriers in the study and to target the intervention towards specific subjects [41]. Before implementing the intervention in large scale, we tested and optimised the intervention in a pilot study among seven general practices to ensure optimal fit with the GPs’ daily clinical practice. The process evaluation followed the recommendations provided by the Medical Research Council (MRC) for evaluation of complex interventions [42]. A detailed description of the pilot study will be published in a separate article.

The intervention consists of a mandatory intervention (for all GPs) and an optional intervention (for GPs that participate in the CME) (Table 1).

**Table 1:** Intervention used in the study; a mandatory component for all GPs and an optional component

	Content	Time
Mandatory intervention	Starting package:	Date of inclusion
	10 iFOBT kits	
	Clinical instruction on iFOBT use in general practice	
	Online educational material	Date of inclusion
	Mail with iFOBT status	Approx. one month after inclusion
Optional intervention	Continuous medical education (CME)	During the first month of inclusion

#### Mandatory intervention

On the date of inclusion, each GP in the included municipality receives a starting package consisting of 10 iFOBT kits and a clinical instruction on iFOBT use in general practice and how to order the test through the online WebReq system. Furthermore, educational material on CRC diagnostics and use of iFOBT will be available on a web page announcing relevant news for GPs (www.praksis.dk). The online material will have links to the slides from the CME PowerPoint presentation, the clinical instruction and images of the contents in the iFOBT kits.

Approx. 1 month after inclusion, participating GPs will receive a mail containing: status on number of tests requested from their municipality, number and rate of positive tests, number of general practices in the municipality that have started using the test and status of the total number of iFOBTs requested in the CDR. Furthermore, information on how to get help to get started is provided.

#### Optional CME intervention

The CME consist of a 45–60 min lecture on CRC diagnostics and use of iFOBT in general practice (Table 2). The CME is an interactive lecture with cases, discussion and questions based on international literature and guidelines and adapted to a Danish general practice setting. GPs attending the meeting will be registered to facilitate suitable grouping of CME-attending general practices.

**Table 2:** Programme of the continuous medical education (CME) of approx. 45–60 min

Welcome
• Introduction of presenters
• Brief introduction to the rational of the study
Presentation, part one: Diagnostics of colorectal cancer in primary care
• Case story
• Update on diagnostics of CRC in today’s general practice
• Why is it important to diagnose CRC in early stages?
Presentation, part two: iFOBT in general practice
• Rational use of iFOBT in general practice
• Indications for using iFOBT
• Actions on test result
• Requesting the test and logistic setup of the study
Questions/discussion
Concluding remarks

### Clinical instruction on iFOBT use in general practice

A clinical instruction was developed for the study (Table 3). The instruction contains suggested indications for using the iFOBT and recommended actions on positive and negative test results. It is aimed for individuals of 30 years or above with symptoms and signs that could be related to CRC. However, iFOBT should not be used on patients presenting alarm symptoms that justify urgent referral to the cancer patient pathway (CPP) [39]. The content is based on published literature.

**Table 3:** Instructions for using iFOBT in general practice (not exhaustive, other indications are possible)

Overall indication	Individuals aged ≥ 30 years with symptoms and signs of colorectal cancer, but do not fulfill the criteria of referral in the CPP
Typical indications	Change in bowel habits^a^
	Abdominal pain^a^
	Anemia or decrease in hemoglobin >10 %^a^
	Diagnostic workup of patients with IBS
	Non-specific symptoms (weight-loss, fatigue, loss of appetite)^b^
Actions on test result	Positive test (≥50 μg/L)
	30–39 years: Referral to colonoscopy with remark of blood in stools found by iFOBT.
	≥40 years: Urgent referral in the cancer patient pathway for colorectal cancer.
	Negative test (≤49 μg/L)
	Colorectal cancer cannot be excluded.

^a^Not eligible for urgent referral in the CPP for CRC

^b^Not eligible for urgent referral in the CPP for non-specific serious symptoms

#### Suggested indications for iFOBT use

Important symptoms and signs of CRC constitute a continuum in general practice [39]. Therefore, iFOBT may be relevant in patients presenting anaemia, change in bowel habits or abdominal pain when these are not eligible for urgent referral. Irritable bowel syndrome (IBS) is generally recommended to be diagnosed using Rome III Criteria with a minimum of diagnostic testing [43, 44]. However, as a positive iFOBT is considered equivalent to rectal bleeding, we found it relevant to recommend performing an iFOBT on patients undergoing evaluation for IBS. Finally, non-specific symptoms such as weight loss, loss of appetite and fatigue are vague symptoms that can be presented in a vast amount of diseases, including different cancer types. Thus, using iFOBT as part of the diagnostic workup of patients presenting non-specific symptoms may aid the GP in the diagnostic process.

#### Recommended actions on positive and negative test results

In this study, the iFOBT is used as a ‘rule in test’. An iFOBT value ≥ 50 μg/L is considered as positive and should be followed by urgent referral to colonoscopy. An iFOBT value ≤ 49 μg/L is considered as negative. As CRC has a low prevalence in general practice, a negative test result should not exclude CRC; a negative result should rather serve to guide the GP in the direction of the most appropriate diagnostic strategy. The iFOBT can also be repeated.

### Analysis of the iFOBT and determination of clinical cut-off

In the Danish screening programme, the iFOBT is analysed on OC-Sensor DIANA (Eiken Chemical Company, Ltd, Japan). In the CDR, the analysis is performed at the Department of Clinical Biochemistry at the Regional Hospital of Randers. All iFOBTs requested from general practice during the study period will be analysed using this existing infrastructure in parallel with the screening samples. iFOBTs are analysed continuously and done by staff blinded to colonoscopic findings.

Studies of iFOBT cut-off values have primarily been conducted in a screening setting [45–49]. The cut-off value in the Danish screening programme is set to 100 μg/L. To our knowledge, no studies have investigated an optimal cut-off value for patients presenting non-alarm symptoms of CRC. Small amounts of blood loss in faeces are normal, but no exact reference level exists [50]. On the other hand, small amounts of blood in faeces may also be indicative of CRC. A low cut-off value for blood in stools increases the number of false positive test results and consequently the number of performed colonoscopies and required resources, whereas a high cut-off increases false negative test results and thereby introduces a risk of delay in the diagnosis [31, 51]. In this study, we set the cut-off value to 50 μg/L. Thus, a value of <50 μg/L will be considered as negative and ≥50 μg/L as positive.

### Logistics

The iFOBTs will be packed in kits together with a patient instruction on how to correctly perform an iFOBT, a paper to facilitate collection of the stool test and a postage-paid envelope addressed to the Regional Hospital of Randers. The packing of iFOBT will be provided by a company with expertise from the screening programme. The iFOBT kits will be delivered to a regional distributor. From here, all GPs will get a box with 10 kits at the date of inclusion. Furthermore, GPs can order additional iFOBT deliveries during the study period. Thus, the access to iFOBT will be easy as the tests will be available in the GPs’ clinics.

Ordering of an iFOBT is done through WebReq, which is an online ordering system used by Danish GPs for requesting laboratory tests. The GP indicates why the iFOBT is required by ticking a simple box in the ordering system. It is possible to tick the indications from the clinical instruction and a space for other symptoms or signs which can be used if the test is requested on other indications. The patient’s iFOBT sample is sent to the Department of Clinical Biochemistry at Randers Regional Hospital for analysis. Test results are returned to the participating GPs electronically and automatically transmitted to the patient’s medical record.

### Outcomes

The uptake and clinical use of iFOBT in general practice

- Frequency of each indication used for requesting iFOBT
  - ○ Indications are registered when the GP orders the test. It is possible to tick the indications from the clinical instruction, and a box for other symptoms.
- Rate and frequency of iFOBT use and characteristics of the patients included.
- The GPs’ action on a positive test result (≥50 μg/L)
  - ○ According to the clinical instruction, a positive iFOBT result should imply referral to colonoscopy.
- The GPs’ action on a negative test result (≤49 μg/L)
  - ○ This outcome will evaluate how patients with a negative test result are followed up.

The diagnostic value of using iFOBT in general practice

- Age- and sex-standardised number and rates of positive tests (>50 μg/L)
- Overall positive predictive value (PPV) for having CRC when iFOBT is positive
- PPV of having CRC in relation to iFOBT cut-off

The clinical implications of using iFOBT in general practice

- Age- and sex-standardised number and rates of urgent referral in the CPP for CRC
- Age- and sex-standardised number and rates of colonoscopies
- All findings of colonoscopy on patients with a positive iFOBT (all ICD-10 codes determined at colonoscopy)
- Age- and sex-standardised number of CRCs diagnosed
- Stage distribution of all CRCs diagnosed (I–IV)

### Data collection

All citizens in Denmark are registered in the Danish Civil Registration System with a unique personal identification number (CPR number). This identification number is used in all national registers and enables accurate linkage between national registers [52]. Statistics Denmark will provide data on socioeconomic and demographic factors [53]. Data on iFOBT value and indications for use of iFOBT will be provided by the Department of Clinical Biochemistry at Randers Regional Hospital through the clinical laboratory information system research database [54]. Data on colonoscopy and comorbidity are extracted from the Danish National Patient Register [55]. Data on CRC diagnosis and disease stage are extracted from the Danish Colorectal Cancer Database.

### Sample size

Each included GP is estimated to request 1–2 iFOBTs per week. This estimate is based on a previous report investigating Danish citizens’ reasons for encounter with a GP [56]. When taking into account that the study is rolled out sequentially and the study period is 1 year, we expect that 33,600 iFOBTs will be performed during the study period. Unpublished data from the Danish screening programme reveal that 6–10 % of performed iFOBTs are positive (≥100 μg/L) depending on age and gender. As this study uses a lower cut-off value (50 μg/L) and the investigated population is symptomatic, we estimate that approx. 10 % of performed tests will be positive. Screening studies have shown that the PPV of having CRC when the iFOBT is positive is approx. 10 % [26, 28, 29]. Therefore, we estimate that 10 % of the patients with a positive test will be diagnosed with CRC. In total, we expect to find approx. 336 CRCs by the use of iFOBT during the study period. Approx. 800 CRCs are annually diagnosed in the CDR. Using an alfa of 0.05 and a power of 0.8, we will be able to show a significant reduction in stage IV cancers from 25 to 16 % of annual CRC. However, as a reduction of this scale is unlikely, the study may be underpowered for this secondary outcome.

### Statistical analysis

The study uses a cluster-randomised stepped-wedge design [40]. Therefore, analysis of data will follow the recommendations for this study design. Each general practice will serve as a control until crossing over to intervention. As iFOBT is not available to Danish GPs before they are included in the study, it will not be possible to compare the use before and after inclusion. However, we will be able to evaluate the dissemination of the test in general practice.

#### Evaluating the uptake and clinical use of iFOBT in general practice

The frequency of each indication used to order the iFOBT will be assessed using descriptive statistics. The development in rate and frequency of iFOBT use are assessed descriptively by illustrating the dissemination with the relation between time and use of iFOBT. One-way ANOVA is used to test for differences in the development of iFOBT use among different municipalities and among different general practices within each municipality. To facilitate comparison of the general practices, they are divided into: clinics where all GPs attended the CME (all-CME-clinics), clinics where at least one, but not all GPs, attended the CME (colleague-CME-clinics) and clinics where no GPs attended the CME (no-CME-clinics). Actions taken on positive test results are assessed by investigating if patients with a positive iFOBT have been referred to colonoscopy. Actions taken on negative test result are assessed by estimating the rate of patients with a negative test result that are referred for colonoscopy and/or has iFOBT repeated.

#### Estimating the diagnostic value of iFOBT in general practice

The total number of performed iFOBTs is assessed, and the number and rates of positive tests are calculated. The overall PPV for CRC in case of iFOBT values ≥50 μg/L is calculated, and the optimal cut-off value for the use of iFOBT on patients presenting non-alarm symptoms of CRC will be investigated by ROC curves using cut-off intervals of 50 μg/L.

#### Investigating the clinical implications of using iFOBT in general practice

Age- and sex-standardised number and rates of colonoscopies and urgent referrals in the CPP are estimated before and after intervention. Findings by colonoscopy are identified by ICD-10 codes, and the PPV of finding serious bowel disease is calculated. Serious bowel disease is defined as: CRC, inflammatory bowel disease or adenomas >1 cm. The number of CRCs diagnosed in the study period is compared with the number of CRCs diagnosed before introducing iFOBT in general practice. The same comparison is done for stage distribution of CRC.

## Discussion

To our knowledge, this is among the first and largest controlled studies on the use of iFOBT in general practice. The study will investigate the implementation, diagnostic value and clinical implications of using iFOBT on patients presenting non-alarm symptoms that could origin from a CRC. The study is implemented stepwise in the CDR among 836 GPs in 381 general practices. An intervention has been developed to optimise implementation. This study may be an important step towards improving the diagnostics of CRC in primary care and detecting CRC in earlier stages.

Only a few small-scale studies have investigated the use of iFOBT in general practice, and the general conclusion was that iFOBT may be useful as a diagnostic tool in general practice [31–37]. However, most studies have included both alarm symptoms and non-alarm symptoms in the evaluation of the test. As many countries have implemented CPPs to improve CRC diagnostics, good diagnostic opportunities already exist for patients presenting with alarm symptoms. However, the CPPs generally seem to prolong the diagnostic process for patients presenting with non-alarm symptoms [57]. On the other hand, all patients presenting symptoms that could originate from a CRC cannot be referred to colonoscopy. Therefore, this study explores the implementation of iFOBT for patients presenting with non-alarm symptoms of CRC.

We found it important to develop a clinical instruction to direct the use of iFOBT in patients presenting with non-alarm symptoms of CRC. We acknowledge that the clinical assessment performed by the GP should guide the exact use. Therefore, the indications presented in the instruction are suggestions. This implies that GPs can request iFOBT for other symptoms or signs that they may find relevant. This gives us the opportunity to explore if the GPs’ needs for the test are in line with the indications in the clinical instruction. The criteria for urgent referral in a CPP are evidently not definite, eg change in bowel habits can be of different levels of severity and could be a symptom of many different diseases. Furthermore, the threshold for referring patients on the basis of a given alarm symptom might be lower for some GPs than for others. Consequently, we decided to include anaemia, change in bowel habits and abdominal pain in the clinical instruction. This is supported by a recent update of the guideline provided by the National Institute for Health and Care Excellence (NICE) [58]. However, it is important to underline that performance of the iFOBT in this study only consider individuals who are not eligible for urgent referral in the CPP for CRC. Giving GPs the possibility of performing iFOBT on ‘low-risk-but-not-no-risk’ patients may result in more complete and timely diagnostic workup.

Using a cluster-randomised stepped-wedge study design allows inclusion of all GPs in the CDR. Using municipalities as clusters implies that all GPs in a given municipality are included at the same time. Thereby, we prevent that some GPs in the municipality are able to order the test, while others are not. Furthermore, this setup provides the opportunity of arranging the CME at a meeting for all GPs in the municipality. This allows GPs to meet and discuss the study with colleagues from their own clinical environment.

Using iFOBT in patients who present non-alarm symptoms of CRC may imply faster and earlier diagnosis. This study constitutes a thorough large-scale investigation of iFOBT in a real-life setting in general practice. The study design enables generalisability to other primary-care settings and will nationally and internationally be very important in deciding future recommendations for diagnostic workup of patients presenting symptoms of CRC.

## Abbreviations

CDR, Central Denmark Region; CME, Continuous medical education; CPP, Cancer patient pathway; CRC, Colorectal cancer; DSAM, Danish College of General Practitioners; gFOBT, Guaiac faecal occult blood test; GP, General practitioner; IBS, Irritable bowel syndrome; iFOBT, Immunochemical faecal occult blood test; NICE, National Institute for Health and Care Excellence; PLO, Organisation of General Practitioners in Denmark; PPV, Positive predictive value
